# Ergonomics and performance of using prismatic loupes in simulated surgical tasks among surgeons – a randomized controlled, cross-over trial

**DOI:** 10.3389/fpubh.2023.1257365

**Published:** 2024-01-09

**Authors:** Xuelong Fan, Liyun Yang, Nathalie Young, Ilayda Kaner, Magnus Kjellman, Mikael Forsman

**Affiliations:** ^1^Department of Medical Sciences, Uppsala University, Uppsala, Sweden; ^2^Institute of Environmental Medicine, Karolinska Institutet, Stockholm, Sweden; ^3^Department of Molecular Medicine and Surgery, Karolinska Institutet, Stockholm, Sweden; ^4^Division of Ergonomics, School of Engineering Sciences in Chemistry, Biotechnology and Health (CBH), KTH Royal Institute of Technology, Huddinge, Sweden

**Keywords:** prismatic loupes, workload assessment, intervention, surgical ergonomics, inertial measurement unit, electromyography

## Abstract

**Introduction:**

Recently developed prismatic loupes may mitigate the high physical workload and risk of neck disorders associated with traditional surgical loupes among surgeons. However, research in this area, particularly among surgeons, is sparse. This study examines the impact of prismatic loupes on surgeons’ physical workload, musculoskeletal discomfort, and performance during simulated surgical tasks.

**Materials and methods:**

Nineteen out of twenty recruited surgeons performed three tasks in a fixed-order with their own loupes and both low-tilt (LT) and high-tilt (HT) prismatic loupes, in a randomized order. The primary outcomes were the median inclination angles and velocities of the head, trunk, and upper arms, along with the median muscle activity of the cervical erector spinae (CES), upper trapezius (UT), and lumbar erector spinae (LES) for each pair of loupes. The secondary outcomes included performance (completion time and errors), perceived body-part discomfort, and subjective evaluation of the three pairs of loupes.

**Results:**

Using prismatic loupes, either LT or HT, compared with the surgeons’ own loupes yielded lower head inclinations (all *p* < 0.001), lower neck muscle activity (all *p* < 0.05), and lower neck discomfort in indirect comparisons (*p* < 0.01) with no significant difference in surgical errors (***p*** = 0.628). However, HT loupes resulted in a longer task completion time in two tasks (*p* < 0.001). Most surgeons preferred LT loupes (*N* = 12) for their comfort and visual functions.

**Discussion:**

The results indicate that prismatic loupes can reduce physical workload in the neck during simulated surgical task, with no significant difference in surgical errors. Future studies are needed to investigate the long-term effects of prismatic loupes among surgeons.

## 1 Introduction

Work-related musculoskeletal disorders (WMSDs) are prevalent among surgeons, irrespective of their surgical modalities and specialties (1–6). Among these, the neck is frequently cited as a common site of severe pain (3–8). When compared to other modalities, surgeons performing open surgeries are particularly vulnerable, exhibiting higher postural loads in the head/neck region (8, 9) and increased muscle activity (10), both of which are critical risk factors for neck and shoulder musculoskeletal disorders (11).

As demonstrated in a previous study, among other factors such as high neck bending angles and precision work requirements, the weight of surgical loupes adds to the torque that impacts in the forward-bending neck and conjointly amplifies the cervical loading (12). However, surgical loupes are essential for many surgeons (12), with 14% reporting daily usage in a previous study among surgeons across various specialties (6). Therefore, there is a need for interventions that reduce surgeon’s cervical workload.

Dentists and dental hygienists work with bending neck and trunk with hands working in a constrained site (13), which is similar to many surgeons, such as those who work in endocrine surgeries (10) and head/neck surgeries. Among dentists and dental hygienists, prismatic glasses have been introduced, which bend the light path downward (14–16). They have been reported to reduce neck bending and lower neck muscle activations, and decrease neck/shoulder discomfort (14–16). However, the angulation of the tested prismatic glasses in previous studies was either4.6° (14, 15) or 90° [estimated, (16)].

The studies on prismatic loupes of low angulation (4.6°) reported significant head and neck flexion reduction among the intervention group in the short-term experiment (14). Though the effects on head and neck flexion reductions were expectedly small (around 5° as the difference between the intervention and the control group), there was still a significant reduction in neck/shoulder pain in the 12-month follow-up study (15). Recommended action levels for limiting the risks of developing MSDs include that the median head flexion should be within 0–25° (17). Previous studies have reported 40°–50° median head flexion among surgeons who perform open surgeries (10, 14). Therefore, if prismatic loupes with low angulation are used to help surgeons improve their working postures and reduce neck flexion, the low angulation of 4.6° in the aforementioned loupes may not be sufficient. Higher angulations may be needed to offer greater ergonomic benefits.

The prismatic loupes with a high angulation (90°), were reported to decrease the head tilt by 20°, which was an equivalent 0° head flexion, border-line the limit of the action level range; this may lead to over-correction or even backward extension of the head (16). The study also reported low preference for the prismatic loupes with a 90° angulation among five tested dental hygienists (16). The low preference may be due to the short experiment period, but more studies are needed to investigate whether prismatic loupes have any limitations with high angulations. As surgeons from certain modalities may share similar working postures, an angulation of 90° may be too extreme if such loupes are introduced for surgeons.

Overall, it is worthwhile to study the effects of prismatic loupes with varying angulations on physical workload, discomfort, and workability.

Despite of the recent development of commercially available prismatic loupes for surgeons [e.g., (18)], studies of the effects of such loupes/glasses on physical workload and health benefits among surgeons are still rare. One study reported adequate correction in head flexion (with a median visually estimated as 15°) with surgical loupes with an angulation of 90° (19). However, the demographical information about the three participants was scarce, and, given the large inter-subject variance among people, the sample size (*N* = 3) of the existing study was small. Further, since only one camera was used to assess neck flexion in real surgeries, the accuracy was likely much lower than in studies using technical measurements. Additionally, the results from the existing study with those reported among dental workers (16, 19). For example, while both studies using prismatic loupes with a 90-degree angulation, the study among dental workers (16) reported reduced productivity, while the study among surgeons did not find significantly decreased productivity (19).

Lastly, it has been reported that the usage of surgical loupe is associated with increased neck/shoulder muscle activation (20). The forward-bending head creates a torque on the neck that makes the muscular load in the neck sensitive to additional weight on the head such as loupes and head light. The angulation of the prismatic loupes can change the tilt of head, ergo the torque. Therefore, it would be beneficial to investigate the relation between neck/shoulder muscle activation and the angulation of the prismatic loupes.

Hence, further studies are needed to investigate the feasibility of using prismatic loupes to reduce surgeons’ physical workload and to evaluate the effects of different angulations of the loupes. This study aimed to compare two types of new prismatic loupes with traditional loupes, regarding surgeons’ physical workload, perceived physical discomfort, surgical performance and subjective evaluation in simulated surgical tasks.

## 2 Materials and methods

### 2.1 Participants

Twenty surgeons with at least 2 years of experience in endocrine, head and neck, or vascular surgeries were recruited from a Swedish academic hospital; the effective sample size was 19 due to dropouts. The selection of surgical specialties was based on the similarity in surgical postures and the use of loupes. Due to the limited availability of eligible participants, the sample size was determined to the fullest extent that the circumstances allowed. Information regarding age, sex, stature, weight, and surgical experience was collected. Each participant was examined by an optician. After the experiment, each participant was provided with one pair of prismatic loupes of their choice, which they can continue using in their daily work. The study was approved by the Regional Ethics Review Board in Stockholm (Dnr: 2020–02161, extended from Dnr: 2014/1120–31). The trial was registered under the number ISRCTN34385943 in the ISRCTN registry. All participants gave their informed consent to participate in the study.

### 2.2 Surgical loupes

Three types of loupes were used in this study: the surgeons’ own traditional nonprismatic loupes (own) with a typical magnification power of 2.5, low-tilt (LT) prismatic loupes (Optergo AB, Mölnlycke, Sweden) with a 15° angulation of the optical axis in the prism and magnification power of 3.0, and high-tilt (HT) prismatic loupes (HOYA Technosurgical, Tokyo, Japan) with an angulation of 48° in the prism and magnification power of 2.5. The LT loupes were custom-made and custom-adjusted for each participant, and the frame provided a fixed forward inclination of 20°. In comparison, the HT loupes were ready-made (not custom-made) with an adjustable inclination angle of the frame up to 30° and were adjusted to fit each participant’s needs before the laboratory trial.

### 2.3 Study design and settings

This randomized, controlled crossover study was conducted at a clinical training center with an individually adjusted surgical light and table height; surgeons adjusted the light and height according to their habits and likings. The participants performed three representative simulated surgical tasks, peg transfer (PT), basic suture (BS), and precision cutting (PC) which were depicted in details in the Supplement 1 and a previous study (21), with each of the three pairs of loupes. The order of the loupes was randomly chosen and balanced from the following four combinations by researchers using the blocking method with a block size of 4: (1) own - > HT - > LT; (2) own - > LT - > HT; (3) HT - > LT - > own; (4) LT - > HT - > own. Given that the focus of the comparison was on the differences between the loupes, the task order was not randomized (PT - > BS - > PC). By using a blocking method and a fixed task order, the number of groups can be limited to a manageable size, i.e., 4. The impact of task sequence on workload measurements was trivial because no outcomes were compared between tasks; all three tasks can be considered as a collective meta-task. Before the experiment day, participants had at least 15 min to familiarize themselves with all three loupes and the three tasks.

### 2.4 Primary outcome measures

The primary outcome measures were the physical workload of the surgeons, including postures and angular velocities of the head, trunk, and upper arms, and the muscular activity of the cervical erector spinae (CES), upper trapezius (UT), and lumbar erector spinae (LES).

Postural data of the head, trunk, and upper arms were recorded by four inertial measurement units (IMUs) (AX6, Axivity Ltd., Newcastle, UK) at 25 Hz in the same positions as a previous study (10), i.e., back of the head (head), mid-point of the sternum (trunk), and under the deltoid (upper arms). For the head and the trunk, the postures were defined as the sagittal inclination angle relative to a neutral posture during which the participant stood upright with eyes looking forward (22). The left and right arms’ postures were defined as any inclination angle relative to a neutral posture when the participant sat in a chair and leaned to the side with a 2-kg dumbbell in hand (23). The IMU data were processed by a Kalman filter to obtain inclination angles and computed according to a previously published study (22). The angular velocities were calculated as the derivatives of the corresponding inclination angles (22).

**Figure 1:**
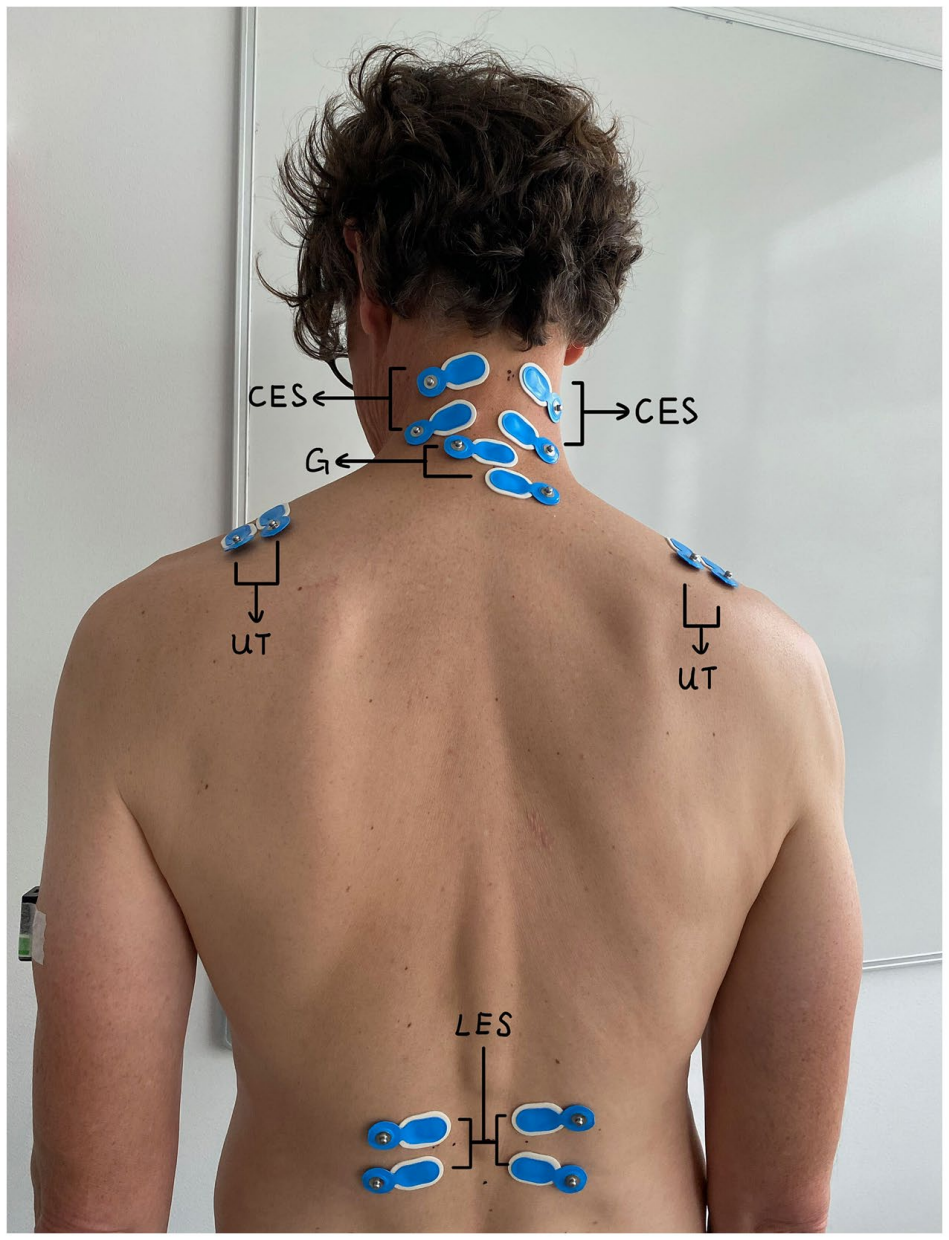
Positions of EMG electrodes on the cervical erector spinae, upper trapezius, and lumbar erector spinae.

The muscle activities were recorded bilaterally by surface electromyography (EMG) using bipolar electrodes with a gel (Ag/AgCl electrodes, N-00-S/25, Ambu A/S, Copenhagen, Denmark) and a logger (Mobi8, from TMSi, Oldenzaal, The Netherlands) with a sampling rate of 1,024 Hz per channel and a 24-bit AD-convertor. The electrodes for the CES were placed bilaterally, with one at the C2–C3 level on the upper part of the trapezius and the other 3 cm caudally (24). For the UT, the electrodes were placed 2 cm lateral to the midpoint from the C7 vertebra to the acromion process with a center-center distance of 2 cm (25). For the LES, the electrodes were placed on the muscle belly 2 cm lateral to the spinous process of L3 (26) with a center-center distance of 3 cm (27). Since two loggers were used, two electrodes were used for the ground; they were placed under and above C7 (See Figure 1).

To obtain muscle activity, the EMG data were first computed as root mean square (RMS) values of every 1/8 s epoch after a digital bandpass filter (30–400 Hz) (28) and then normalized as percentages of maximal voluntary electrical activation (%MVE), or reference voluntary electrical activation (%RVE). The MVEs were obtained individually during three maximal voluntary contractions (MVCs) for the CES and the UT, and the RVEs for LES were obtained during reference voluntary contractions (RVCs) with a 1-min rest between the contractions for each muscle (26, 28). To perform the MVCs for the CES, participants extended their necks maximally and statically against their hands on the back of their head; for the UT, participants held their arms at 45 degrees and resisted downward pressure from a researcher on the upper arm. To measure the RVCs of the LES, participants lay on a bench, lifted their upper torso over the edge, and held for 5 s while a researcher stabilized their feet.

### 2.5 Secondary outcome measures

The perceived visual quality, body-part discomfort, and subjective evaluation of the user experience was evaluated by a survey after the trial of each loupe and at the end of the whole experiment.

The perceived visual quality was measured by a survey that contains indirect and direct comparison after the use of each pair of loupes. For the indirect comparison, a 5-point Likert-type scale (from –2, very bad, to +2, very good) was employed to measure the overall visual function, image brightness, spatial orientation, and image depth, and a 4-point scale (from –3, severe, to 0, nothing) was used for assessing double vision, headache, and nausea (14, 21). For the direct comparison, the surgeons were asked, after all the trials, to rank the three loupes (from 1, the best, to 3, the worst) regarding all aforementioned aspects. Equal ranks were allowed in situations where no difference could be sensed.

The perceived discomfort was assessed by Borg’s CR-10 scale for the neck, right shoulder, left shoulder, upper back, and lower back (29, 30).

Subjective evaluation of the user experience contained two parts – a single-choice question on the preferred loupes and an open question asking for any comments on the three pairs of loupes were included (see Supplement 4).

Additionally, surgical performance was assessed by task completion time and counting the number of surgical errors. The errors were rated by two independent surgeon examiners. An error was counted when the participant missed one dot or did not go through one dot in the suture for BS and when the participant cut outside the black line for PC. Errors were not counted in PT.

### 2.6 Data processing and statistical analysis

The postures and muscle activities were summarized as the 10th, 50th, and 90th percentiles of all data within each task, with each pair of loupes for each participant. For the angular velocities, only the 50th percentile was used (17).

Equal ranks in the final survey were normalized so that the sum of all ranks remained at 6, e.g., a rank of [1, 1, 2] would be normalized to [1.5, 1.5, 3]. The number of surgical errors was calculated as the average value of the two examiners.

All quantitative measures of each task were compared separately across three types of loupes (own, LT, and HT), and they were matched on the individual level. The normality and sphericity of the dataset of each measure were checked by the Shapiro–Wilk test and Mauchly’s test. Since the normality and the sphericity of most measures did not fulfill the criteria, the Friedman test was used to test the overall significance of the differences in each measure of each task between the three pairs of loupes (own, LT, and HT). *Post hoc* analyses were conducted using the Wilcoxon signed-rank test with the Bonferroni correction when the Friedman test results were significant. The alpha level was chosen as 0.05. Open-question results were analyzed with thematic analysis.

## 3 Results

### 3.1 Participants

Twenty surgeons were recruited for this study from September to December 2021. One surgeon was excluded from the analysis due to double vision with the HT loupes. The demographics of the 19 included participants are shown in Table 1.

**Table 1:** Demographics of participants.

		Participants
Characteristics		(*n* = 19)
Sex, *N* (%)		
Male		12 (63)
Female		7 (37)
Age, median (IQR), y		49 (45–53)
Statue, median (IQR), cm		176.0 (166.5–183.5)
BMI, median (IQR), kg/m^2^		24.2 (21.2–26.7)
Handedness, *N* (%)		
Right-handed		17 (89)
Left-handed		1 (5)
Completely ambidextrous		1 (5)
Experience as a surgeon, *N* (%)		
6–10 years		5 (26)
11–15 years		2 (11)
16–20 years		5 (26)
20+ years		7 (37)
Specialty, *N* (%)		
Vascular surgery		3 (16)
Ear, nose, and throat surgery		8 (42)
Endocrine surgery		8 (42)
Frequency of using only surgical loupes, *N* (%)		
Never		3 (16)
At least once per month		2 (11)
At least once per week		3 (16)
At least once per day		1 (5)
Always		10 (53)
Frequency of using both loupes and headlamp, *n* (%)		
Never		3 (16)
At least once per month		3 (16)
At least once per week		3 (16)
At least once per day		0 (0)
Always		10 (53)

### 3.2 Postural workload of head, trunk, and arms

**Figure 2:**
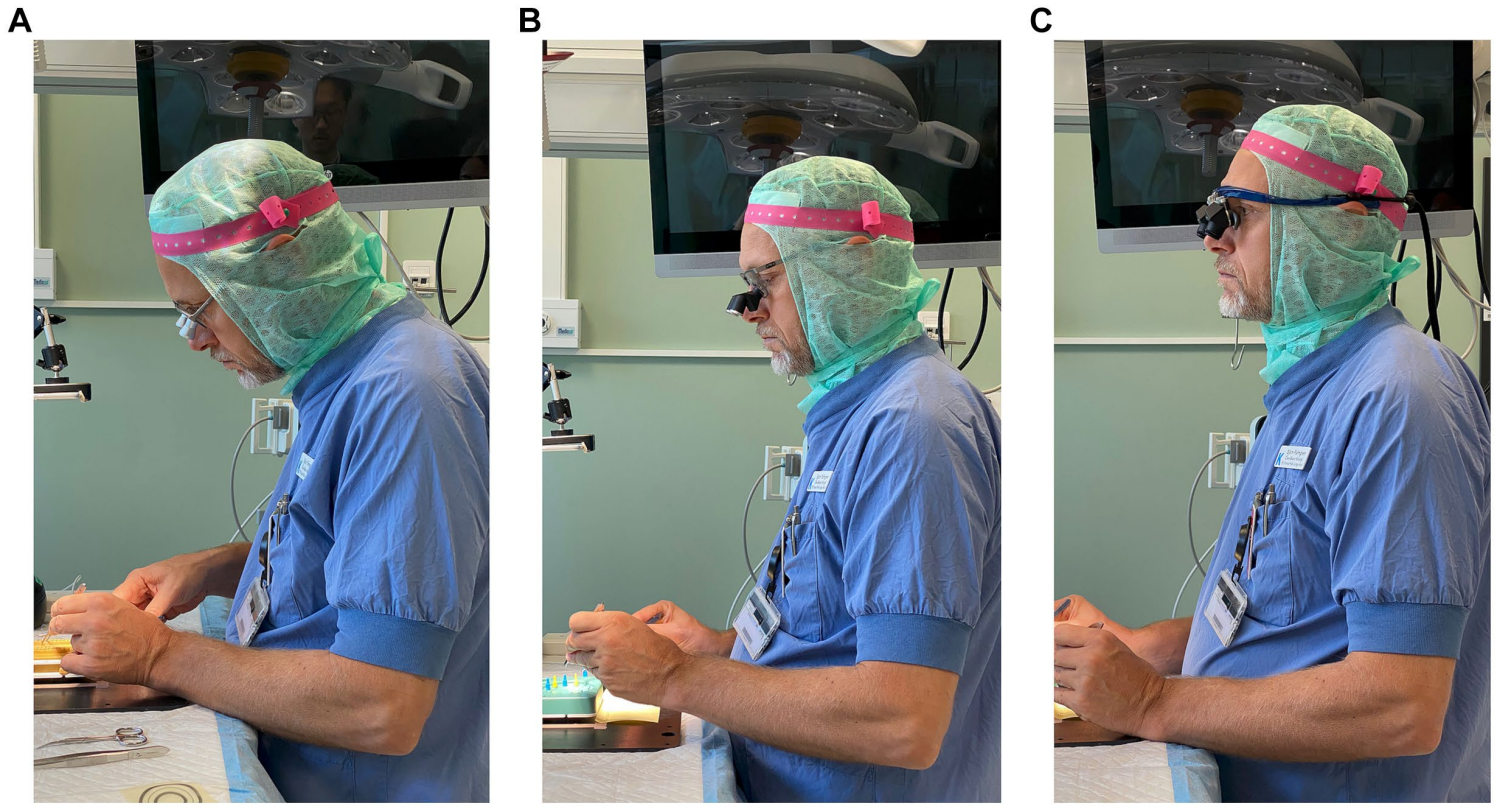
A typical side-view of the posture when using three different loupes. **(A–C)** are the surgeons’ own loupes, LT prismatic loupes, and HT prismatic loupes, respectively.

**Figure 3:**
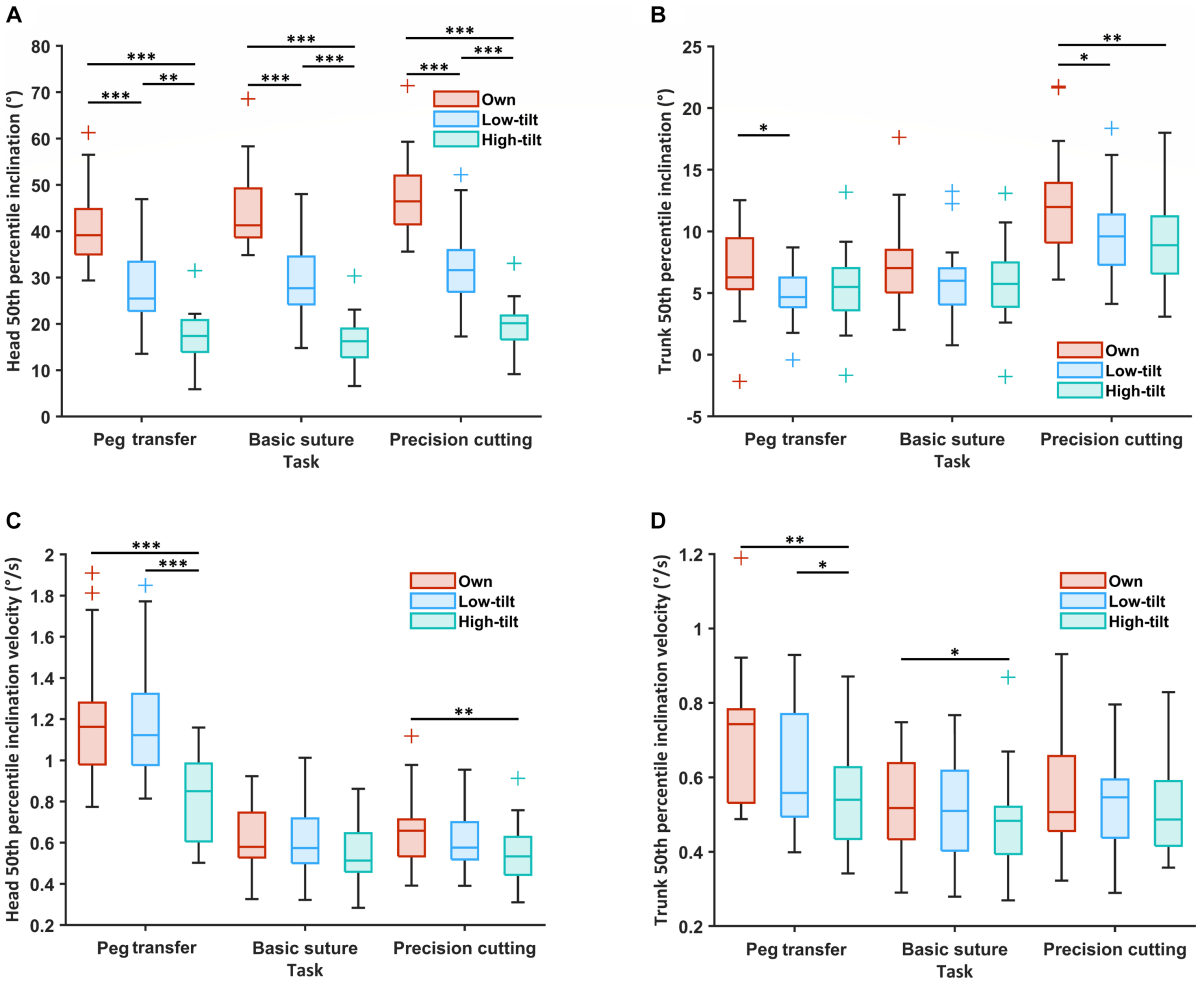
The postural workload of the 19 participants, including the inclination angle (50th percentile; °) in **(A,B)** and velocity (50th percentile; °/s) in **(C,D)** of the head **(A,C)** and trunk **(B,D)** during three simulated surgical tasks, compared among the surgeons’ own surgical loupes, the low-tilt loupes, and the high-tilt loupes. For each box, the middle line represents the median value; the upper and lower edges of the box denote the upper and lower quartiles; the upper edge and lower edge of the whiskers indicate the nonoutlier maximum and minimum, and outliers are marked with +. Significant differences are denoted with ^*^ when *p* < 0.05, ^**^ when *p* < 0.01, and ^***^ when *p* < 0.001.

Figure 2 shows the typical postures surgeons had when using three different loupes. From the surgeons’ own loupes to the LT loupes and the HT loupes, the 50th percentile of the head inclination angle decreased significantly in all three tasks (°, median [IQR]): PT [own loupes, 39 (35–45); LT, 25 (23–33); HT, 17 (14–21); all *p* < 0.001], BS [own, 41 (39–49); LT, 28 (24–35); HT, 16 (13–19); all *p* < 0.001], and PC [own, 46 (41–52); LT, 32 (27–36); HT, 20 (17–22); all *p* < 0.001] (Figure 3). The trunk and arm inclination angles were similar across different loupes in the three tasks, except for a slight but significant decrease in the trunk angle from surgeons’ own loupes to the LT and HT loupes during PC (Supplement 2).

When using the high-tilt (HT) loupes, the right-arm inclination velocity was slightly, yet significantly, lower compared to when using the other two types of loupes in all three tasks [°/s, median (IQR)]: PT [median (IQR), own, 1.9 (1.5–2.4); LT, 1.7 (1.0–3.0); HT 1.3 (0.9–1.9); HT vs. own, *p* = 0.001; HT vs. LT, *p* = 0.006], BS [own, 0.9 (0.8–1.3); LT, 1.0 (0.7–1.3); HT 0.8 (0.7–0.9); HT vs. own, *p* = 0.001; HT vs. LT, *p* = 0.001], and PC [own, 1.3 (1.1–1.9); LT, 1.3 (1.0–1.8); HT 1.0 (0.9–1.6); HT vs. own, *p* = 0.003; HT vs. LT, *p* = 0.002]. In some tasks, a lower inclination velocity for the HT loupes was also found for the head, trunk, and left arm (Supplement 2).

### 3.3 Muscle activity of the cervical erector spinae, upper trapezius, and lumbar erector spinae

**Figure 4:**
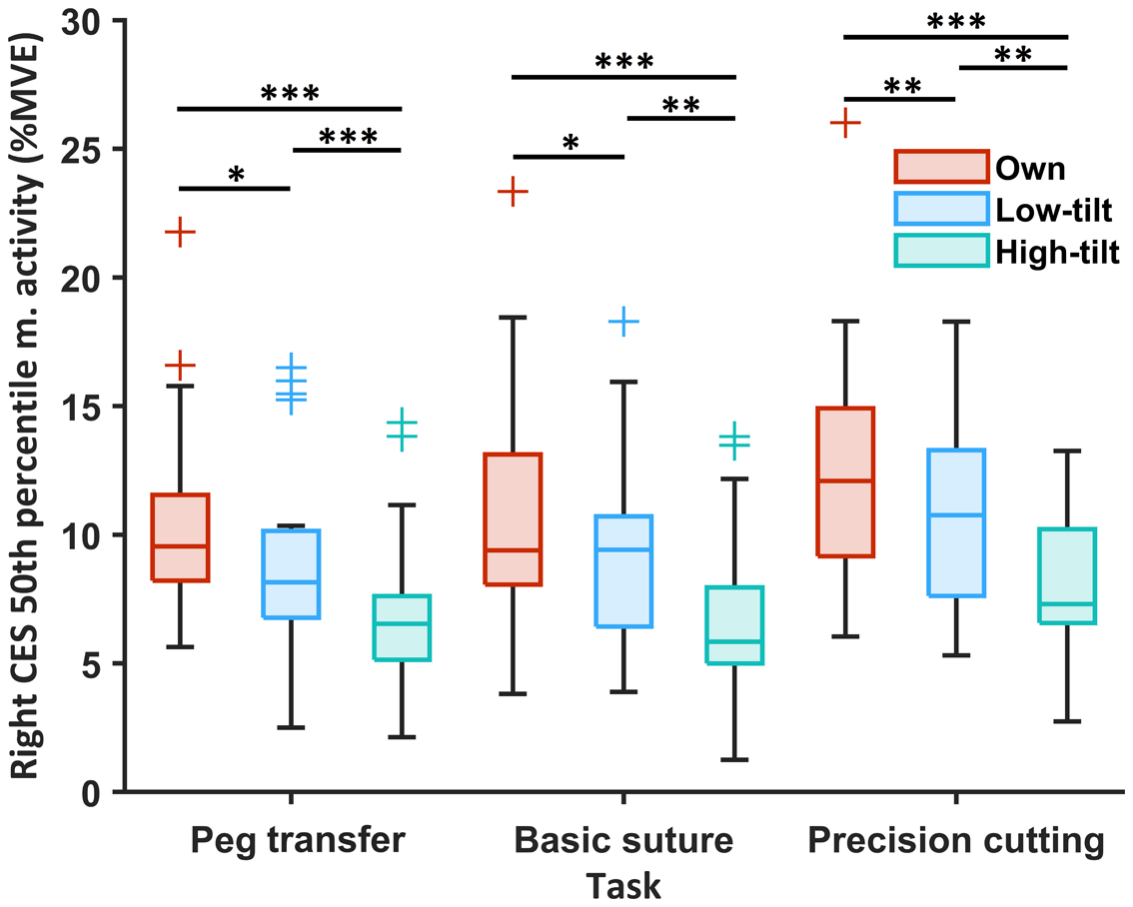
Muscle activity (50th percentile; %MVE) of the right CES of the 19 participants during three simulated surgical tasks compared among the surgeons’ own surgical loupes, the low-tilt loupes, and the high-tilt loupes. For each box, the middle line represents the median value; the upper and lower edges of the box denote the upper and lower quartiles; the upper edge and lower edge of the whiskers denote the nonoutlier maximum and minimum; and outliers are marked with +. Significant differences are indicated with ^*^ when *p* < 0.05, ^**^ when *p* < 0.01, and ^***^ when *p* < 0.001.

From the surgeons’ own loupes to LT loupes and HT loupes, the 50th percentile of the muscle activity of the right CES decreased significantly in all three tasks [%MVE, median (IQR)]: PT [own, 9.5 (8.2–11.5); LT, 8.2 (6.8–10.1); HT 6.5 (5.1–7.6); own vs. LT, *p* = 0.047; own vs. HT, *p* < 0.001; LT vs. HT, *p* < 0.001], BS [own, 9.4 (8.1–13.1); LT, 9.4 (6.4–10.7); HT 5.8 (5.0–7.9); own vs. LT, *p* = 0.021; own vs. HT, *p* < 0.001; LT vs. HT, *p* = 0.001], and PC [own, 12.1 (9.2–14.9); LT, 10.8 (7.6–13.3); HT 7.3 (6.6–10.2); own vs. LT, *p* = 0.009; own vs. HT, *p* < 0.001; LT vs. HT, *p* = 0.002] (Figure 4). Such a significant reduction in muscle activity was also observed in the left CES across all tasks and in the left and right UT or LES in a few tasks (Supplement 3).

### 3.4 Perceived visual quality and perceived discomfort

The LT loupes were reported to have better image brightness [median (IQR)] than the surgeons’ own loupes both in the indirect comparison [own, 1.0 (0.3–1.0); LT, 1.0 (1.0–2.0); own vs. LT, *p* = 0.046] and in the direct comparison [own, 2.5 (1.1–3.0); LT, 1.0 (1.0–1.9); own vs. LT, *p* = 0.019]; however, no significant differences were found in any other examined perceived visual quality, i.e., the overall visual function, spatial orientation, image depth, double vision, headache, or nausea (Table 2). In comparison to using their own loupes, the participants reported significantly lower neck discomfort [median (IQR)] when using LT and HT loupes in the indirect comparison [own, 1.0 (0.0–2.0); LT, 0.0 (0.0–1.0); HT 0.0 (0.0–0.9); own vs. LT, *p* = 0.006; own vs. HT, *p* = 0.006], but no significant differences were found in the indirection comparison of the reported shoulder or back discomfort. The differences in the direct comparison of neck discomfort were insignificant, though there was a trend of lower discomfort ratings for the prismatic loupes (Table 2). No significant differences were found in the other compared variables regarding the perceived visual quality and perceived discomfort.

**Table 2:** Survey results after each loupe (*N* = 19).

Comparison	Question	Own	Low-tilt	High-tilt	adjusted value of p			
		median [IQR]	median [IQR]	median [IQR]	Friedman test	Own vs. LT	Own vs. HT	LT vs. HT
*Scale − 2 to + 2 (very bad, bad, neutral, good, very good)*								
Indirect	Overall visual function	1.0 [1.0–1.8]	1.0 [1.0–2.0]	1.0 [0.3–1.8]	0.069	–	–	–
	Image brightness	1.0 [0.3–1.0]	1.0 [1.0–2.0]	1.0 [1.0–2.0]	**0.006**	**0.046**	0.059	>0.999
	Spatial orientation	1.0 [1.0–2.0]	1.0 [1.0–2.0]	1.0 [0.0–1.0]	0.106	–	–	–
	Image depth	1.0 [1.0–1.0]	1.0 [1.0–2.0]	1.0 [0.0–1.8]	0.212	–	–	–
*Scale − 3 to 0 (severe, moderate, mild, no)*								
Indirect	Double vision	0.0 [0.0–0.0]	0.0 [0.0–0.0]	0.0[0.0–0.0]	0.449	–	–	–
	Headache	0.0 [0.0–0.0]	0.0 [0.0–0.0]	0.0[0.0–0.0]	0.223	–	–	–
	Nausea	0.0 [0.0–0.0]	0.0 [0.0–0.0]	0.0[−0.8–0.0]	**0.041**	>0.999	0.137	0.137
*Scale 0 to 10 (nothing at all to very, very hard (maximal))*								
Indirect	Neck discomfort	1.0 [0.0–2.0]	0.0 [0.0–1.0]	0.0[0.0–0.9]	**0.001**	**0.006**	**0.006**	0.396
	Right shoulder discomfort	0.0 [0.0–1.0]	0.0 [0.0–0.5]	0.0[0.0–0.8]	0.326	–	–	–
	Left shoulder discomfort	0.0 [0.0–0.5]	0.0 [0.0–0.0]	0.0[0.0–0.0]	0.513	–	–	–
	Upper back discomfort	0.0 [0.0–1.0]	0.0 [0.0–0.0]	0.0[0.0–0.0]	**0.016**	0.182	0.077	>0.999
	Low back discomfort	0.0 [0.0–0.9]	0.0 [0.0–0.0]	0.0[0.0–0.0]	**0.006**	0.069	0.102	0.952
*Rank 1 to 3 (best to worst)*								
Direct	Overall visual function	2.0 [1.1–3.0]	1.5 [1.0–2.4]	2.0 [1.6–3.0]	0.214	–	–	–
	Image brightness	2.5 [1.1–3.0]	1.0 [1.0–1.9]	1.0 [1.0–2.0]	**0.009**	**0.019**	0.062	>0.999
	Spatial orientation	2.0 [1.0–2.0]	2.0 [1.0–2.0]	3.0 [1.0–3.0]	0.201	–	–	–
	Image depth	1.5 [1.0–2.4]	1.5 [1.0–2.0]	2.0 [1.1–3.0]	0.256	–	–	–
	Double vision	1.0 [1.0–1.5]	1.0 [1.0–1.5]	1.0 [1.0–1.9]	0.661	–	–	–
	Headache	1.0 [1.0–1.0]	1.0 [1.0–1.0]	1.0 [1.0–2.0]	0.401	–	–	–
	Nausea	1.0 [1.0–1.5]	1.0 [1.0–1.4]	1.0 [1.0–2.9]	**0.048**	>0.999	0.134	0.070
	Neck discomfort	2.5 [1.0–3.0]	1.0 [1.0–1.9]	1.0 [1.0–1.9]	**0.012**	0.115	0.065	>0.999
	Right shoulder discomfort	1.0 [1.0–2.8]	1.0 [1.0–1.4]	1.0 [1.0–1.9]	0.239	–	–	–
	Left shoulder discomfort	1.0 [1.0–2.6]	1.0 [1.0–1.4]	1.0 [1.0–1.4]	0.241	–	–	–
	Upper back discomfort	1.0 [1.0–1.4]	1.0 [1.0–1.0]	1.0 [1.0–1.0]	0.337	–	–	–
	Low back discomfort	1.0 [1.0–1.0]	1.0 [1.0–1.0]	1.0 [1.0–1.0]	0.211	–	–	–

*p* values are adjusted by Bonferroni correction; bold *p* values are significant (*p* < 0.05).

### 3.5 Subjective evaluation

Most participants preferred LT loupes (5 for own; 11 for LT; 2 for HT; 1 for own or LT depending on the surgery case; 1 for “own loupes for now or HT if double vision can be fixed”). For the LT loupes, nine participants stated that they liked the visual functions of the loupes because of their good spatial orientation (*N* = 2), brightness (*N* = 2), image sharpness (*N* = 2), and the benefits of having glasses around the loupes, which provide good side vision (*N* = 4). Seven participants stated that the LT loupes were comfortable because of their lightweight (N = 2) and good fit (N = 5). Three participants stated they had a satisfactory posture when using the LT loupes. However, three participants reported double vision or vision distortion with the LT loupes, and two stated that the LT loupes had a smaller field of vision.

Regarding the HT loupes, five participants mentioned feeling “relaxed/relieved” in the neck. Six participants stated that the HT loupes had good visual functions, including clear vision (*N* = 2), a bright image (*N* = 4), and a broad field of vision (*N* = 4). In contrast, eight participants were dissatisfied with the visual functions of the HT loupes, mentioning reasons including a feeling of dizziness (*N* = 3), double vision (N = 2), shadows in the view (*N* = 2), and a difference in sharpness within the view (*N* = 1). In addition, two participants mentioned the disadvantage of the HT loupes not having glasses around the loupes for side vision and as a shield against splashed blood.

Regarding their own loupes, four participants stated that they were used to their own loupes and that they fit well. While four participants expressed that their own loupes caused them to have a bad posture, three mentioned that their own loupes were old and needed visual adjustment.

### 3.6 Performance of surgeons

**Figure 5:**
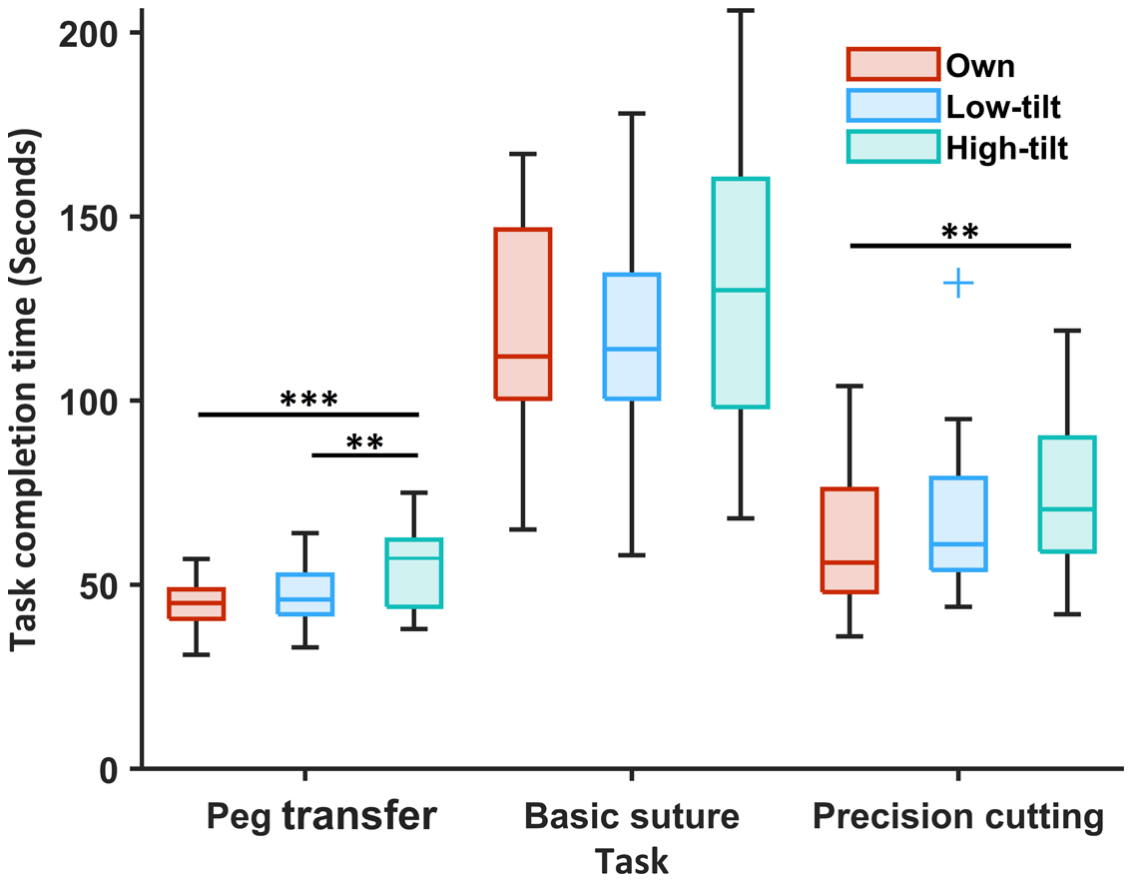
Task completion time of three simulated surgical tasks of the 19 participants compared among the surgeons’ own surgical loupes, the low-tilt loupes, and the high-tilt loupes. For each box, the middle line represents the median value; the upper and lower edges of the box denote the upper and lower quartiles; the upper edge and lower edge of the whiskers denote the nonoutlier maximum and minimum; and outliers are marked with +. Significant differences are indicated with ^*^ when *p* < 0.05, ^**^ when *p* < 0.01, and ^***^ when *p* < 0.001.

The task completion time [seconds, median (IQR)] was significantly longer when using the HT loupes in the PT compared to the surgeons’ own loupes and the LT loupes [own, 45 (41–49); LT, 46 (42–53); HT 57 (44–62); LT vs. HT, *p* = 0.002; own vs. HT, *p* < 0.001], and in the PC compared to the surgeon’s own loupes [own, 56 (48–76); HT 71 (59–90); own vs. HT, *p* = 0.005] (Figure 5). There were no significant differences in the numbers of surgical errors between different loupes in the BS [own, 0.5 (0.0–1.0); LT, 0.5 (0.0–1.4); HT 1.0 (0.0–1.5); overall, *p* = 0.628] or the PC [own, 0.5 (0.0–1.4); LT, 1.0 (0.1–1.5); HT 0.5 (0.0–1.0); overall, *p* = 0.404].

## 4 Discussion

The main results of this randomized crossover study were that using prismatic loupes (compared to traditional loupes) reduced two ergonomic risk factors – head inclination and neck muscle activity –without increasing surgical errors. However, in two of three tasks, the completion time was prolonged when using HT loupes.

To our best knowledge, this is currently the largest performed study that focuses on the effects of prismatic loupes use among surgeons. There was a significant reduction in head inclination when using the two prismatic loupes compared to traditional loupes. This is an important finding since the forward head inclination likely is a primary contributor to the high frequency of neck/shoulder pain among surgeons. An angulation of 15° in the prism of the LT loupes resulted in a 13°–14° reduction in the group median of the 50th percentile head inclination, while a 48° angulation in the prism of the HT loupes reduced the head inclination by 22°–26°. While factors such as eye rolling and frame angle also influence the impact of loupes on head inclination, the results consistently show a trend: increased angulation in the prism of the surgical loupes correlates with greater head inclination.

A few studies have reported that the usage of prismatic glasses can reduce head or neck flexion among dental workers, with an angulation angle of 5° in the prism leading to a reduction of 6.5° in the 50th percentile head inclination (14) and an angulation of 90° resulting in a 33°–39° decrease in the 50th percentile head tilt (16). Another study found that among surgeons, 90°-angulated prismatic glasses reduced head inclination by 35° (19). These results show that using prismatic glasses improves head posture. A proposed action level for a whole-day measurement of median head inclination has been set at 25° (17). Although the median levels of head inclination in this experimental study with task times of a few minutes cannot be directly compared to that proposed at the whole workday action level, the observed significant reduction from 39°–46° to 17°–32° suggests that the usage of prismatic loupes can decrease the risks of MSDs.

This study also found that in comparison to that with the use of nonprismatic loupes, the neck muscle activity significantly decreased on both sides when using prismatic loupes, with a 0–23% reduction in the group median of the 50th percentile muscle activity for the LT loupes and a 32–42% reduction for the HT loupes. This reduction was smaller than in a previous study (~40% reduction in the group mean of the 10th percentile), which used loupes with a 90-degree angulation for a simulated dental task (16). Both results suggest that prismatic loupes can reduce muscle activity in the neck. Further experience of using the new loupes may lead to further reduction.

Regarding surgical performance, no significant difference in surgical errors was found between the nonprismatic loupes and the LT and HT prismatic loupes. However, it took the participants longer to complete the activities in two of the three simulated tasks with the HT loupes than with the nonprismatic loupes and LT loupes, though the practice session provided for all participants before the trials were short. Another finding was that the upper arms were slightly more static when the surgeons used the HT loupes than the others. In the previous study by Smith et al. (16) with participants with no prior experience in dental work, in comparison to seeing the object directly, using prismatic glasses (90° angulation) decreased the accuracy and productivity in simulated dental procedures. The results from this and the previous study indicate that high-tilt prismatic loupes/glasses may negatively impact work performance. It is worth mentioning that both studies used laboratory-based simulated tasks and provided little training time; hence, the results can only indicate short-term impacts of the prismatic loupes; if the prismatic loupes are used for a longer time, these impacts are likely to decrease. Concerning long-term health effects, a one-year cohort study showed improved workability among dental personnel when using prismatic glasses (15). This has been supported by the preliminary results from a field study with a more extended training period (90 min) with prismatic glasses. In that study, no significant differences in the completion time of surgeries were observed (19). Future studies should examine the effects of prismatic loupes on surgery performance, including surgery completion time, after sufficient training or use of the loupes.

Reduced physical workload is related to reduced risks of developing MSDs (11, 17). Two previous studies, one in dentistry and one in cleft palate surgery (with the latter including only three surgeons), performed comparisons with and without glasses (i.e., without amplification) with a 90° angulation in the prism (16, 19). Both studies revealed a significant decrease in neck discomfort directly after the tasks or during the short term (<1 day). In this study, in comparison to the nonprismatic loupes, both prismatic loupes yielded significantly lower neck discomfort in the indirect comparison. This difference was, however, not significant in the direct comparison and only showed a trend of reduced comfort ratings. This ambiguity of results between the indirect and the direct comparison may be explained by the short experiment time and the narrower range of scales used in the direct comparison. The former explanation is supported by a series of two studies regarding prismatic glasses with a 5° angulation for dental personnel, of which the first study, with a 9–11 week follow-up time, did not find a significant difference in neck comfort or neck exertion when comparing prismatic glasses to nonprismatic loupes (14). However, a significant difference in neck exertion and neck pain was found in the second, long-term (12-month) study (15). Further investigations are needed to determine the long-term effects of prismatic loupes on the prevalence of MSDs among surgeons.

It is important to note that factors other than angulation in the prism should also be considered when choosing or designing prismatic loupes. The higher angulation can lead to significantly lower head forward inclination angles, but the user experience of such loupes might, on the other hand, be worse than those with slightly lower angulation. This is revealed by the results that LT loupes were preferred by most surgeons in this study, while HT loupes were less preferred. The comfort, e.g., “lightweight and fit,” visual quality, and the presence of glasses around the loupe for side vision during surgery were important factors contributing to the surgeons’ preference for LT prismatic loupes in addition to the resultant neck posture. For the future design of prismatic surgical loupes, finding the most suitable angulation for different types of surgeries is worth further studying.

## 5 Limitations

One limitation is that due to one drop-out and missing data caused by lost contact of electrodes, a perfect balance between groups of the original study design was not achieved, especially for the EMG data. Two of the four missing data sets for EMG regarded LES, and they were related to the two prismatic loupes. However, most measured variables were not impacted by the missing data, and the amount of missing data comprised only a small portion of the total amount of data. Previous studies (14, 19) found a paired difference around 4° and 30° (estimated) with a standard deviation of 5° and 11°, which yields a required sample size of around 16 or 3. The sample size in this study was at least 17 and mostly 19, which means the sample size of available data is still sufficient to support the results. Another limitation is that the study only investigated head flexion, which includes both trunk and neck flexion. Nevertheless, the head flexion can still reflect the surgeons’ posture, and the observed reduction of the head flexion with the prismatic loupes is still meaningful. Thirdly, there was a difference in the magnification of the two prismatic loupes and their level of customization. Therefore, the difference in the outcomes of the two prismatic loupes may stem from factors other than the angulation in the prism, such as the tilt angle of the frames and the range of eye movement, which is also related to the magnification of the lenses. As a result, angulation angles should be chosen carefully along with the other factors in the design of prismatic loupes. This study indicated the advantages of prismatic loupes, but more studies are needed to investigate other design factors. Lastly, the results were based on simulated tasks performed within a short period in a laboratory setting with limited training time, especially concerning the prismatic loupes, which the surgeons had not used before the study. The laboratory setting with simulated work tasks was considered necessary before introducing the new prismatic loupes to surgeons for use in actual operations. Still, under such short exposure time with the three types of loupes, statistically significant results could be observed in the indirect comparison of neck discomfort. Therefore, the difference may be even more prominent after longer exposure, such as in actual surgeries. The selected tasks reflect typical surgical routines but do not cover all scenarios. However, they do generally align with the advised patient orientation on the operating table to promote ergonomic posture for the surgeon. Future investigations should examine the effects of prismatic loupes in actual surgery among surgeons with longer practice times.

## 6 Conclusion

Compared to traditional loupes, this study shows that both evaluated prismatic loupes can significantly reduce neck muscle activity and forward head bending in surgical tasks, with no significant difference in surgical errors. Nevertheless, using the HT prismatic loupes prolonged the task completion times, but this was only after a short training period. LT loupes were preferred by a majority of the surgeons in this study. The significant results from this study are in favor of the usage of prismatic loupes in reducing the surgeon’s workload. Future studies are needed to investigate the extent to which prismatic loupes may decrease physical workloads and reduce musculoskeletal discomfort among surgeons in the operating room and over the long term. In addition, the design of prismatic surgical loupes should consider multiple factors, including not only the prism angulation but also factors such as magnification, peripheral vision, and comfort to suit surgeons’ needs for different types of surgeries.

## Data availability statement

The raw data supporting the conclusions of this article will be made available by the authors, without undue reservation.

## Ethics statement

The studies involving humans were approved by The Regional Ethics Review Board in Stockholm. The studies were conducted in accordance with the local legislation and institutional requirements. The participants provided their written informed consent to participate in this study. Written informed consent was obtained from the individual(s) for the publication of any potentially identifiable images or data included in this article.

## Author contributions

XF: Conceptualization, Data curation, Formal analysis, Funding acquisition, Investigation, Methodology, Project administration, Visualization, Writing – original draft, Writing – review & editing. LY: Formal analysis, Investigation, Methodology, Writing – original draft, Writing – review & editing. NY: Data curation, Writing – review & editing. IK: Data curation, Writing – review & editing. MK: Conceptualization, Funding acquisition, Methodology, Resources, Supervision, Writing – review & editing. MF: Conceptualization, Funding acquisition, Methodology, Resources, Supervision, Writing – review & editing.
