# Supplementary material for: Ergonomics and performance of using prismatic loupes in simulated surgical tasks among surgeons – a randomized controlled, cross-over trial
Source: Front Public Health. 2024 Jan 9;11:1257365. doi: 10.3389/fpubh.2023.1257365 (PMC10803506; doi:10.3389/fpubh.2023.1257365)
Supplement: Supplementary file 1 [file Table_1.docx]

## **Supplement 2**

## eTable 1. Physical workload of head, trunk, and arms (n=19, if not specified otherwise). P-values are adjusted by Bonferroni correction; bold p-values are significant.

| Task | Body section | Measure | Own | Low-tilt | High-tilt | adjusted p-value | | | |
| --- | --- | --- | --- | --- | --- | --- | --- | --- | --- |
|  |  |  | median[IQR] | median[IQR] | median[IQR] | Friedman test | Own vs LT | Own vs HT | LT vs HT |
| *Inclination angle (°)* | | | | | | | | | |
| Peg transfer | Head | 10th | 37[33–43] | 24[21–31] | 16[12–19] | **<0.001** | **<0.001** | **<0.001** | **0.001** |
|  |  | 50th | 39[35–45] | 25[23–33] | 17[14–21] | **<0.001** | **<0.001** | **<0.001** | **0.001** |
|  |  | 90th | 42[38–48] | 29[26–36] | 19[15–22] | **<0.001** | **<0.001** | **<0.001** | **<0.001** |
|  | Trunk | 10th | 6[3–8] | 4[3–5] | 4[2–6] | 0.076 | - | - | - |
|  |  | 50th | 6[5–9] | 5[4–6] | 5[4–7] | **0.003** | **0.013** | 0.133 | 0.442 |
|  |  | 90th | 7[6–10] | 6[5–7] | 7[5–8] | **0.003** | **0.011** | 0.192 | 0.477 |
|  | Left arm | 10th | 15[10–18] | 14[10–19] | 14[11–16] | 0.504 | - | - | - |
|  |  | 50th | 17[14–20] | 15[11–21] | 15[13–18] | 0.810 | - | - | - |
|  |  | 90th | 22[16–26] | 21[13–27] | 20[15–26] | 0.229 | - | - | - |
|  | Right arm | 10th | 16[12–17] | 16[13–20] | 16[13–17] | 0.854 | - | - | - |
|  |  | 50th | 18[15–23] | 20[16–26] | 17[16–23] | 0.331 | - | - | - |
|  |  | 90th | 23[20–34] | 27[21–38] | 23[20–31] | 0.150 | - | - | - |
| Basic suturing | Head | 10th | 40[36–47] | 26[22–33] | 14[11–18] | **<0.001** | **<0.001** | **<0.001** | **<0.001** |
|  |  | 50th | 41[39–49] | 28[24–35] | 16[13–19] | **<0.001** | **<0.001** | **<0.001** | **<0.001** |
|  |  | 90th | 42[40–51] | 29[26–36] | 17[14–21] | **<0.001** | **<0.001** | **<0.001** | **<0.001** |
|  | Trunk | 10th | 5[4–8] | 5[3–6] | 5[3–6] | 0.692 | - | - | - |
|  |  | 50th | 7[5–8] | 6[4–7] | 6[4–7] | 0.143 | - | - | - |
|  |  | 90th | 8[6–10] | 7[5–8] | 6[5–8] | 0.080 | - | - | - |
|  | Left arm | 10th | 12[8–18] | 13[9–17] | 13[8–19] | 0.076 | - | - | - |
|  |  | 50th | 13[9–19] | 14[11–20] | 15[9–20] | 0.229 | - | - | - |
|  |  | 90th | 15[13–23] | 17[14–21] | 16[12–22] | 0.229 | - | - | - |
|  | Right arm | 10th | 19[15–22] | 19[13–22] | 18[14–21] | 0.241 | - | - | - |
|  |  | 50th | 21[17–24] | 22[16–24] | 20[16–22] | 0.368 | - | - | - |
|  |  | 90th | 24[20–27] | 25[19–29] | 23[20–27] | 0.431 | - | - | - |
| Precision cutting | Head | 10th | 42[39–50] | 29[25–34] | 18[15–20] | **<0.001** | **<0.001** | **<0.001** | **<0.001** |
|  |  | 50th | 46[41–52] | 32[27–36] | 20[17–22] | **<0.001** | **<0.001** | **<0.001** | **<0.001** |
|  |  | 90th | 49[45–55] | 35[29–39] | 22[20–26] | **<0.001** | **<0.001** | **<0.001** | **<0.001** |
|  | Trunk | 10th | 9[5–11] | 7[4–8] | 6[5–7] | **0.008** | **0.006** | **0.015** | >0.999 |
|  |  | 50th | 12[9–14] | 10[7–11] | 9[7–11] | **0.004** | **0.016** | **0.005** | >0.999 |
|  |  | 90th | 16[14–24] | 12[10–16] | 12[11–15] | **0.003** | **0.004** | **0.004** | >0.999 |
|  | Left arm | 10th | 11[6–13] | 13[9–18] | 13[6–15] | **0.014** | **0.024** | 0.379 | 0.514 |
|  |  | 50th | 14[9–22] | 16[10–20] | 15[10–20] | 0.692 | - | - | - |
|  |  | 90th | 19[14–25] | 20[15–24] | 19[13–25] | 0.854 | - | - | - |
|  | Right arm | 10th | 17[14–20] | 18[15–22] | 16[14–21] | 0.268 | - | - | - |
|  |  | 50th | 28[26–31] | 27[25–32] | 27[22–30] | 0.331 | - | - | - |
|  |  | 90th | 46[45–54] | 46[42–52] | 43[41–51] | 0.331 | - | - | - |
| *Inclination velocity (°/s)* | | | | | | | | | |
| Peg transfer | Head | 50th | 1.2[1.0–1.3] | 1.1[1.0–1.3] | 0.9[0.6–1.0] | **<0.001** | >0.999 | **<0.001** | **<0.001** |
|  | Trunk |  | 0.7[0.5–0.8] | 0.6[0.5–0.8] | 0.5[0.4–0.6] | **0.008** | 0.192 | **0.003** | **0.024** |
|  | Left arm |  | 1.4[0.5–1.9] | 1.2[0.6–1.6] | 0.8[0.4–1.1] | **<0.001** | >0.999 | **<0.001** | **<0.001** |
|  | Right arm |  | 1.9[1.5–2.4] | 1.7[1.0–3.0] | 1.3[0.9–1.9] | **0.002** | >0.999 | **0.001** | **0.006** |
| Basic suturing | Head |  | 0.6[0.5–0.7] | 0.6[0.5–0.7] | 0.5[0.5–0.6] | **0.012** | 0.886 | 0.053 | 0.099 |
|  | Trunk |  | 0.5[0.4–0.6] | 0.5[0.4–0.6] | 0.5[0.4–0.5] | **<0.001** | 0.409 | **0.016** | 0.176 |
|  | Left arm |  | 0.7[0.6–1.0] | 0.7[0.6–0.9] | 0.6[0.5–0.9] | **0.018** | >0.999 | 0.081 | **0.008** |
|  | Right arm |  | 0.9[0.8–1.3] | 1.0[0.7–1.3] | 0.8[0.7–0.9] | **<0.001** | >0.999 | **0.001** | **0.001** |
| Precision cutting | Head |  | 0.7[0.5–0.7] | 0.6[0.5–0.7] | 0.5[0.4–0.6] | **0.014** | 0.210 | **0.002** | 0.099 |
|  | Trunk |  | 0.5[0.5–0.7] | 0.5[0.4–0.6] | 0.5[0.4–0.6] | 0.229 | - | - | - |
|  | Left arm |  | 0.6[0.4–0.7] | 0.5[0.4–0.6] | 0.4[0.3–0.6] | **0.006** | 0.379 | **0.016** | **0.024** |
|  | Right arm |  | 1.3[1.1–1.9] | 1.3[1.0–1.8] | 1.0[0.9–1.6] | **0.001** | >0.999 | **0.003** | **0.002** |
| ^a^ n=17; two are missing due to technical reasons | | | | | | | | | |
| ^b^ n=18; one is missing due to technical reasons | | | | | | | | | |
