# Supplementary material for: Ergonomics and performance of using prismatic loupes in simulated surgical tasks among surgeons – a randomized controlled, cross-over trial
Source: Front Public Health. 2024 Jan 9;11:1257365. doi: 10.3389/fpubh.2023.1257365 (PMC10803506; doi:10.3389/fpubh.2023.1257365)
Supplement: Supplementary file 2 [file Table_2.docx]

# **Supplement 3**

## eTable 2. Muscle activity of cervical erector spinae (%MVE), upper trapezius (%MVE), and lumbar erector spinae (%RVE) (n=19, if not specified otherwise). P-values are adjusted by Bonferroni correction; bold p-values are significant.

| Task | Body section | Measure | Own | Low-tilt | High-tilt | adjusted p-value | | | | |
| --- | --- | --- | --- | --- | --- | --- | --- | --- | --- | --- |
|  |  |  | median [IQR] | median [IQR] | median [IQR] | Friedman test | Own vs LT | Own vs HT | LT vs HT |  |
| Peg transfer | Left CES | 10th | 5.7 [4.3–7.4] | 5.0 [4.0–6.1] | 3.5 [3.0–5.4] | **<0.001** | **0.030** | **0.001** | **0.015** |  |
|  |  | 50th | 8.1 [5.7–9.9] | 6.7 [5.4–8.3] | 4.8 [4.2–6.9] | **<0.001** | **0.006** | **0.001** | **0.013** |  |
|  |  | 90th | 10.8 [7.4–12.8] | 8.8 [7.0–11.8] | 6.3 [5.5–9.2] | **<0.001** | **<0.001** | **0.001** | **0.011** |  |
|  | Right CES | 10th | 7.0 [6.1–8.5] | 5.8 [4.8–7.4] | 4.6 [3.7–6.0] | **<0.001** | 0.059 | **<0.001** | **<0.001** |  |
|  |  | 50th | 9.5 [8.2–11.5] | 8.2 [6.8–10.1] | 6.5 [5.1–7.6] | **<0.001** | **0.047** | **<0.001** | **<0.001** |  |
|  |  | 90th | 12.3 [10.3–15.1] | 11.4 [9.2–13.2] | 9.1 [6.6–11.7] | **<0.001** | **0.015** | **<0.001** | **<0.001** |  |
|  | Left trapezius | 10th | 1.1 [0.2–2.6] | 0.4 [0.2–3.1] | 0.7 [0.2–2.5] | 0.331 | - | - | - |  |
|  |  | 50th | 2.5 [0.4–3.9] | 1.1 [0.3–4.3] | 1.3 [0.4–4.1] | 0.504 | - | - | - |  |
|  |  | 90th | 3.8 [2.3–6.5] | 3.0 [0.8–6.1] | 2.6 [1.3–6.4] | 0.692 | - | - | - |  |
|  | Right trapezius | 10th | 1.6 [0.5–3.9] | 0.8 [0.4–3.7] | 0.7 [0.4–3.2] | 0.692 | - | - | - |  |
|  |  | 50th | 2.8 [0.9–5.8] | 3.4 [1.1–5.7] | 2.1 [0.8–5.1] | 0.949 | - | - | - |  |
|  |  | 90th | 6.6 [2.9–8.5] | 6.5 [2.0–8.5] | 3.2 [2.0–7.7] | 0.854 | - | - | - |  |
|  | Left LES | 10th | 8.5 [4.8–13.6]^a^ | 9.4 [4.2–15.7]^a^ | 7.8 [4.8–13.6]^a^ | 0.465 | - | - | - |  |
|  |  | 50th | 14.2 [9.4–22.9]^a^ | 14.9 [7.2–20.6]^a^ | 13.0 [7.8–17.9]^a^ | 0.101 | - | - | - |  |
|  |  | 90th | 17.5 [13.2–29.3]^a^ | 19.4 [11.8–25.5]^a^ | 17.3 [12.0–22.1]^a^ | 0.101 | - | - | - |  |
|  | Right LES | 10th | 12.2 [5.2–16.2]^a^ | 11.6 [8.5–16.3]^a^ | 9.9 [6.5–15.5]^a^ | 0.230 | - | - | - |  |
|  |  | 50th | 18.3 [11.2–23.3]^a^ | 16.4 [13.0–21.0]^a^ | 16.9 [12.5–21.6]^a^ | 0.291 | - | - | - |  |
|  |  | 90th | 23.5 [16.4–28.4]^a^ | 20.7 [18.0–26.5]^a^ | 21.0 [16.7–26.9]^a^ | 0.390 | - | - | - |  |
| Basic suturing | Left CES | 10th | 7.0 [4.4–7.8] | 5.2 [4.1–6.5] | 3.9 [3.1–4.7] | **<0.001** | **0.011** | **<0.001** | **<0.001** |  |
|  |  | 50th | 9.1 [5.8–10.2] | 7.0 [5.2–8.6] | 5.3 [4.0–5.9] | **<0.001** | **0.003** | **<0.001** | **<0.001** |  |
|  |  | 90th | 11.3 [7.4–12.7] | 8.9 [6.5–10.6] | 6.5 [5.2–8.1] | **<0.001** | **<0.001** | **<0.001** | **<0.001** |  |
|  | Right CES | 10th | 7.3 [6.3–10.1] | 6.5 [4.8–8.4] | 4.6 [3.1–6.5] | **<0.001** | **0.047** | **<0.001** | **<0.001** |  |
|  |  | 50th | 9.4 [8.1–13.1] | 9.4 [6.4–10.7] | 5.8 [5.0–7.9] | **<0.001** | **0.021** | **<0.001** | **0.001** |  |
|  |  | 90th | 13.2 [9.7–16.4] | 11.7 [8.3–13.8] | 7.6 [6.5–10.0] | **<0.001** | **0.002** | **<0.001** | **<0.001** |  |
|  | Left trapezius | 10th | 1.6 [0.5–2.9] | 0.9 [0.3–3.0] | 0.9 [0.3–2.6] | 0.179 | - | - | - |  |
|  |  | 50th | 2.3 [1.3–4.7] | 1.9 [0.9–3.9] | 1.5 [0.8–3.5] | **0.029** | 0.379 | 0.120 | 0.099 |  |
|  |  | 90th | 3.8 [2.0–6.3] | 3.1 [1.6–5.5] | 2.8 [1.5–4.6] | **0.040** | 0.477 | 0.133 | 0.099 |  |
|  | Right trapezius | 10th | 2.9 [0.7–4.5] | 2.4 [0.7–4.2] | 1.1 [0.4–2.7] | **0.016** | >0.999 | **0.011** | **0.027** |  |
|  |  | 50th | 4.3 [1.2–5.8] | 4.0 [1.5–5.7] | 1.8 [0.8–4.3] | **0.004** | >0.999 | **0.007** | **0.024** |  |
|  |  | 90th | 6.4 [2.0–7.7] | 5.4 [2.9–7.5] | 3.3 [1.5–7.2] | **0.006** | >0.999 | **0.008** | **0.013** |  |
|  | Left LES | 10th | 10.7 [4.6–12.7]^a^ | 7.7 [3.9–12.9]^a^ | 8.8 [3.8–13.8]^a^ | 0.494 | - | - | - |  |
|  |  | 50th | 15.2 [8.9–19.3]^a^ | 12.0 [7.1–17.5]^a^ | 11.5 [8.2–18.3]^a^ | **0.035** | 0.408 | 0.148 | >0.999 |  |
|  |  | 90th | 18.4 [13.1–25.1]^a^ | 16.4 [10.3–23.3]^a^ | 15.7 [11.7–22.4]^a^ | **0.035** | 0.372 | 0.133 | >0.999 |  |
|  | Right LES | 10th | 14.0 [8.3–19.7]^a^ | 12.9 [7.8–15.6]^a^ | 12.8 [7.8–14.3]^a^ | 0.161 | - | - | - |  |
|  |  | 50th | 18.2 [14.7–25.7]^a^ | 18.2 [10.6–20.6]^a^ | 16.8 [11.1–19.9]^a^ | **0.014** | 0.307 | 0.050 | 0.094 |  |
|  |  | 90th | 23.2 [18.5–31.3]^a^ | 22.6 [13.9–25.9]^a^ | 20.3 [13.8–24.9]^a^ | **0.035** | 0.629 | 0.050 | 0.118 |  |
| Precision cutting | Left CES | 10th | 7.1 [5.1–8.3]^b^ | 6.1 [4.7–7.4]^b^ | 4.5 [3.9–6.0]^b^ | **<0.001** | **0.017** | **<0.001** | **0.001** |  |
|  |  | 50th | 10.0 [6.6–11.2]^b^ | 8.3 [6.4–9.9]^b^ | 6.1 [5.4–8.8]^b^ | **<0.001** | **0.001** | **<0.001** | **0.002** |  |
|  |  | 90th | 13.6 [8.7–14.5]^b^ | 10.7 [7.9–13.8]^b^ | 7.9 [7.1–11.5]^b^ | **<0.001** | **0.004** | **<0.001** | **0.002** |  |
|  | Right CES | 10th | 8.9 [6.6–11.7]^b^ | 8.0 [5.9–9.5]^b^ | 5.5 [4.9–7.2]^b^ | **<0.001** | **0.004** | **<0.001** | **0.002** |  |
|  |  | 50th | 12.1 [9.2–14.9]^b^ | 10.8 [7.6–13.3]^b^ | 7.3 [6.6–10.2]^b^ | **<0.001** | **0.009** | **<0.001** | **0.002** |  |
|  |  | 90th | 16.8 [13.6–19.2]^b^ | 14.4 [9.6–17.5]^b^ | 9.8 [9.1–13.9]^b^ | **<0.001** | **0.010** | **<0.001** | **0.009** |  |
|  | Left trapezius | 10th | 2.7 [1.3–3.3] | 1.6 [0.7–3.2] | 1.4 [0.6–2.5] | 0.065 | - | - | - |  |
|  |  | 50th | 3.7 [2.0–5.2] | 2.4 [1.5–4.7] | 2.3 [1.3–3.7] | **0.016** | 0.730 | **0.010** | 0.176 |  |
|  |  | 90th | 5.7 [4.7–7.9] | 4.7 [3.3–6.6] | 4.9 [2.2–5.5] | **0.014** | 0.514 | **0.005** | 0.073 |  |
|  | Right trapezius | 10th | 5.1 [2.4–5.5] | 3.6 [2.3–5.2] | 2.7 [1.2–5.0] | **0.004** | 0.943 | **0.015** | **0.022** |  |
|  |  | 50th | 7.4 [6.0–9.1] | 6.7 [5.3–9.3] | 6.2 [4.9–7.8] | **0.012** | >0.999 | **0.004** | 0.251 |  |
|  |  | 90th | 12.4 [9.5–13.8] | 10.9 [8.3–13.0] | 10.6 [8.1–12.3] | 0.128 | - | - | - |  |
|  | Left LES | 10th | 8.5 [6.7–11.9]^a^ | 7.9 [4.7–12.8]^a^ | 6.8 [3.7–15.4]^a^ | 0.327 | - | - | - |  |
|  |  | 50th | 17.3 [14.2–22.8]^a^ | 16.5 [12.3–22.0]^a^ | 15.5 [8.9–24.1]^a^ | **0.025** | 0.488 | 0.057 | >0.999 |  |
|  |  | 90th | 30.1 [20.9–32.8]^a^ | 23.2 [16.8–32.5]^a^ | 27.0 [14.9–32.3]^a^ | **0.035** | 0.065 | 0.074 | >0.999 |  |
|  | Right LES | 10th | 16.6 [9.3–20.7]^a^ | 13.2 [7.9–15.2]^a^ | 13.1 [9.1–15.6]^a^ | 0.059 | - | - | - |  |
|  |  | 50th | 21.1 [16.9–28.7]^a^ | 18.8 [15.5–24.5]^a^ | 19.0 [13.4–21.4]^a^ | 0.056 | - | - | - |  |
|  |  | 90th | 29.4 [24.2–37.2]^a^ | 25.7 [22.9–35.3]^a^ | 24.3 [21.9–29.4]^a^ | **0.004** | 0.057 | **0.026** | 0.860 |  |
| ^a^ n = 17; two are missing due to technical reasons | | | | | | | | | |  |
| ^b^ n = 18; one is missing due to technical reasons | | | | | | | | | |  |
