# Supplementary material for: Ergonomics and performance of using prismatic loupes in simulated surgical tasks among surgeons – a randomized controlled, cross-over trial
Source: Front Public Health. 2024 Jan 9;11:1257365. doi: 10.3389/fpubh.2023.1257365 (PMC10803506; doi:10.3389/fpubh.2023.1257365)
Supplement: Supplementary file 3 [file Data_Sheet_1.docx]

# **Supplement 1**

# **Description of tasks**

All tasks were conducted on a flat metal surface located on a surgical table, set within a room that replicates an actual operating room. Each task consisted of a separate module, which could be easily attached to or removed from the metal surface. Below are the descriptions of the tasks, each facilitated by one of these removable modules.

### Peg transfer

Participants should use a pair of thumb forceps to transfer 12 pegs that were colored with yellow and blue in an alternating order from reservoirs to 12 pins that were evenly distributed in a circle.

### Basic suture

Participants should run a suture with surgical forceps through 9 pairs of black dots on each side of a simulated wound with a length of 4 cm to close the wound.

### Precision cutting

Participants should use surgical scissors to cut through a 3 mm thick black circle with an inner diameter of 4.7 cm on a fixed skin-like rubber sheet.
