# Supplementary material for: Ergonomics and performance of using prismatic loupes in simulated surgical tasks among surgeons – a randomized controlled, cross-over trial
Source: Front Public Health. 2024 Jan 9;11:1257365. doi: 10.3389/fpubh.2023.1257365 (PMC10803506; doi:10.3389/fpubh.2023.1257365)
Supplement: Supplementary file 4 [file Data_Sheet_2.docx]

# **Supplement 4**

## **Preference question and open questions after all tasks**

1. Which loupes would you prefer out of three? (Choose one of the three)

| - Loupes A | - Loupes B | - Loupes C |
| --- | --- | --- |

1. Do you have any comments regarding those three loupes?*

________________________________________________________________________________________________________________________________________________________________________________________________________________________________________________

* Similar question was also repeated after each pair of loupes and each section of self-reported questions regarding visual ergonomics and musculoskeletal discomfort.
